# Regulation Mechanisms of Expression and Function of Organic Cation Transporter 1

**DOI:** 10.3389/fphar.2020.607613

**Published:** 2021-01-21

**Authors:** Giuliano Ciarimboli

**Affiliations:** Experimental Nephrology, Medicine Clinic D, Münster University Hospital, Münster, Germany

**Keywords:** transporter, regulation, excretion, liver, kidneys

## Abstract

The organic cation transporter 1 (OCT1) belongs together with OCT2 and OCT3 to the solute carrier family 22 (SLC22). OCTs are involved in the movement of organic cations through the plasma membrane. In humans, OCT1 is mainly expressed in the sinusoidal membrane of hepatocytes, while in rodents, OCT1 is strongly represented also in the basolateral membrane of renal proximal tubule cells. Considering that organic cations of endogenous origin are important neurotransmitters and that those of exogenous origin are important drugs, these transporters have significant physiological and pharmacological implications. Because of the high expression of OCTs in excretory organs, their activity has the potential to significantly impact not only local but also systemic concentration of their substrates. Even though many aspects governing OCT function, interaction with substrates, and pharmacological role have been extensively investigated, less is known about regulation of OCTs. Possible mechanisms of regulation include genetic and epigenetic modifications, rapid regulation processes induced by kinases, regulation caused by protein–protein interaction, and long-term regulation induced by specific metabolic and pathological situations. In this mini-review, the known regulatory processes of OCT1 expression and function obtained from *in vitro* and *in vivo* studies are summarized. Further research should be addressed to integrate this knowledge to known aspects of OCT1 physiology and pharmacology.

## Introduction

Organic cations (OCs) are positively charged substances with multiple biological significances as neurotransmitters, metabolic waste products, xenobiotics, and drugs. Their movement through the plasma membrane is mediated by transporters, such as the organic cation transporters (OCTs), which for this reason have important physiological, toxicological, and pharmacological implications. Three OCT paralogs are known: OCT1–3, which work as polyspecific, pH- and Na^+^-independent, bidirectional transporters (Ciarimboli, 2008). OCTs have a species-specific tissue distribution. For example, OCT1 in humans is mainly expressed in the liver (Gorboulev et al., 1997; Nies et al., 2009), while in rodents, it is also present in the kidneys (Jonker and Schinkel, 2004; Holle et al., 2011). Important substances of endogenous and exogenous origin are transported by OCT1. Acetylcholine and monoamine neurotransmitters, and the antidiabetic metformin and the anticholinergic trospium are examples for substrates of endogenous and exogenous origin, respectively (Busch et al., 1996; Wang et al., 2002; Lips et al., 2005; Wenge et al., 2011). Since OCT1 has a high level of expression in excretory organs, its activity has the potential to significantly impact not only local but also systemic concentrations of its substrates. Many aspects of physiology and pharmacology of OCT1 are well known; however, less attention has been paid to regulation processes of this transporter. Therefore, this mini-review is aimed at collecting the information available in the literature about regulation of OCT1 and to underline the possible physiological, pharmacological, and pathological implications of such a regulation.

## Cellular Processing of OCTs

Generally, OCTs localize to the basolateral membrane domain of polarized cells, and, specifically, OCT1 is highly expressed in the sinusoidal membrane of the hepatocytes (Wright and Dantzler, 2004). However, immunohistochemical staining suggested that OCT1 may be also present in the apical domain of the plasma membrane of human renal tubules (Tzvetkov et al., 2009) and enterocytes (Han et al., 2013), suggesting that the localization signals determining polarized expression of OCTs are not inherent to the OCT structure, but probably depend on specific processing mechanisms of the cells, where the transporters are expressed. Human OCT1 (hOCT1), like all the other OCTs, contains potential *N*-linked glycosylation sites in the big extracellular loop (Zhang et al., 1997). This glycosylation may be important for the trafficking of OCTs to the plasma membrane, as demonstrated for rabbit OCT2 (Pelis et al., 2006). Cysteine residues in the big extracellular loop of rat OCT1 (rOCT1) are important for transporter homo-oligomerization, which influences its plasma membrane insertion, without changing the transport characteristics (Keller et al., 2011). In other OCT paralogs, cysteine residues in the extracellular loop seem to have a similar meaning for oligomerization processes and transporter cellular processing (Brast et al., 2012), suggesting that this is a common property of OCTs. Therefore, modifications in this part of OCTs can change their cellular expression pattern and activity.

Another possible mechanism to regulate protein activity derives from a direct interaction with other proteins. Such an interaction may be important for regulation of cellular processing of the transporter, like its trafficking to/from the plasma membrane, and for stabilization of its expression in the plasma membrane. For example, the importance of interaction partners for transporter regulation has been already shown for the Na^+^-glucose cotransporter 2 (SGLT2) and for the Na^+^-dependent neutral amino acid transporter B (0)AT1 in the kidneys. Here, a direct interaction with PDZK1-interacting protein 1 (PDZK1P1, also known as MAP17, a protein mainly expressed in the apical brush border membranes from renal proximal tubules) stimulates SGLT2 activity (Calado et al., 2018), and the interaction of B(0)AT1 with collectrin stabilizes the transporter in the apical plasma membrane of renal proximal tubules (Camargo et al., 2009). Focusing on OCT1, a specific interaction of OCT1 with another protein, may explain why the transporter has a clear basolateral cellular localization in some tissues, while in others, it appears to be expressed on the apical membrane domain. However, there are only few studies aimed at identifying OCT1 interaction partners. A screening performed using the mating-based split-ubiquitin system, a special yeast-two-hybrid technique, able to detect protein–protein interactions taking place in the plasma membrane, identified 24 potential interaction partners for hOCT1 (Snieder et al., 2019). According to gene ontology annotations, the interacting proteins are associated mainly with transport processes, vesicle-mediated transport, signaling pathways, protein modification, homeostatic processes, and cell adhesion (Snieder et al., 2019). The cellular distribution of the identified interaction partners may reflect hOCT1 cellular processing: they are localized in the plasma membrane (CD9 (tetraspanin-29), CYSTM1 (cysteine-rich and transmembrane domain containing protein 1), and PDZKP1), in the endoplasmic reticulum (KRTCAP2 (keratinocyte-associated protein 2), SERP1 (stress-associated endoplasmic reticulum protein 1), VAMPB (vesicle-associated membrane protein-associated protein B isoform 1), and TMEM147 (transmembrane protein 147), in the Golgi system (CHST12 (carbohydrate (chondroitin 4) sulfotransferase 12) and TMBIM4 (transmembrane protein 41B), in endosomes and lysosomes (CD63 (tetraspanin-30) and LAPTM4A (lysosomal associated protein transmembrane 4 α), and in mitochondria (FIS1 (fission, mitochondrial 1 protein), GHITM (growth hormone–inducible transmembrane protein), and SLC25A11 (solute carrier family 25, member 11)). Of special interest may be the hOCT1/PDZK1P1 interaction, which may explain why hOCT1 in the kidneys appears to be expressed in the apical plasma membrane domain (Tzvetkov et al., 2009). Of course, these interactions with hOCT1 should be confirmed using an independent system.

## Short-Term Regulation of Organic Cation Transporter 1 Activity

Substrate transport is one of the main functional performances of the liver and of the kidneys. These organs use transporters to secrete variable quantity of substances, depending on rapidly changing fluid and meal intake and metabolic activities. For this reason, a rapid regulation of hepatic and renal transport functions, adapting their activity to variable situations, is possible. Short-term transporter regulation can be achieved by posttranslational modifications like phosphorylation/dephosphorylation, which can alter transport kinetics. Indeed, both the liver and the kidneys are the target of several hormones, which regulate multiple signaling pathways. For example, insulin regulates in the liver glucose, lipid, and energy metabolism *via* binding to tyrosine kinase receptors, starting a cascade of phosphorylation reactions (Boucher et al., 2014). Conversely, glucagon, by binding to its hepatic receptors, activates adenylate cyclase, which stimulates protein kinase A (PKA) and cyclic AMP (cAMP) response element-binding (CREB) protein. The activation of this pathway leads to increased gluconeogenesis (Janah et al., 2019). In the kidneys, the peptide hormone angiotensin II (Ang II) regulates the most important Na^+^ transporters of the proximal tubules (the apical Na^+^/H^+^ exchanger isoform 3, the basolateral Na^+^-HCO_3_^−^ cotransporter, and the basolateral Na^+^/K^+^-ATPase) in a concentration-dependent manner. For example, at low picomolar to nanomolar concentrations, Ang II stimulates these transporters by binding to the Ang II receptor type 1 (AT1R), activating the protein kinase C (PKC), and/or lowering intracellular cAMP concentration (Shirai et al., 2014).

Rapid regulation has been investigated in cells overexpressing mouse, rat, or human OCT1 (mOCT1 (Schlatter et al., 2014), rOCT1 (Mehrens et al., 2000; Ciarimboli et al., 2005), hOCT1 (Ciarimboli et al., 2004)), and in isolated proximal tubules (PT) from mouse (Holle et al., 2011; Guckel et al., 2012; Schlatter et al., 2014) and rabbit (Hohage et al., 1994) kidneys. In mouse and rabbit PT, both OCT1 and OCT2 are expressed (Kaewmokul et al., 2003; Schlatter et al., 2014); however, OCT1 seems to be the functionally predominant form in mouse PT (Schlatter et al., 2014).

Most studies on acute regulation of OCT1 have been performed measuring the effects of short-time (10 min) activation or inhibition of various kinase pathways on OCT1 orthologs (m, r, or hOCT1) overexpressed in human embryonic kidney (HEK293) cells using the fluorescent organic cation 4-(4-(dimethylamino) styryl-N-methyl-pyridinium (ASP^+^) as a substrate. This technique allows a dynamic measurement of transporter function with high time resolution, as explained in detail elsewhere (Ciarimboli and Schlatter, 2016). In the following, the main results of these studies are summarized.

### Rapid Regulation of mOCT1

Transport mediated by mOCT1 was stimulated by the activity of Ca^2+^–calmodulin complex (CaM), p56^lck^ tyrosine kinase, PKA, and phosphoinositide 3-kinase (PI3K) (Schlatter et al., 2014) (Table 1). Only PKC activation inhibited mOCT1 transport (Schlatter et al., 2014) (Table 1).

**TABLE 1:** Summary of the short-term effects of selected regulation pathways on the organic cation transport mediated by specific organic cation transporter 1 (OCT1) orthologs (mouse (m), rat (r), and human (h) OCT1) in expression systems and in isolated murine proximal tubules (PT) freshly isolated from the kidneys of wild-type (WT) mice and of mice with genetic deletion of OCT2 (OCT2^−/−^) (↑ indicates a stimulation of the transport activity; ↓ indicates an inhibition of the transport activity; 0 indicates no effect on the transport activity). Where measured, the regulation effect on transporter kinetic parameters (affinity, Km; or maximum velocity, Vmax) is also reported.

	Transporter/freshly isolated proximal tubules (PT)				
Activated pathway	mOCT1	rOCT1	hOCT1	OCT2^−/−^mouse PT	WT mouse PT
PKA	**↑** (Schlatter et al., 2014)	**↑** (Mehrens et al., 2000), 0 and **↓** ^*^ (Gerlyand and Sitar, 2009)	**↓** (Ciarimboli et al., 2004); K_*m*_-effect	**↑** (Schlatter et al., 2014)	**↑** (Guckel et al., 2012; Holle et al., 2011; Schlatter et al., 2014); V_*max*_-effect (Guckel et al., 2012)
PKC	**↓** (Schlatter et al., 2014)	**↑** (Gerlyand & Sitar, 2009), (Mehrens et al., 2000); K_*m*_-effect (Mehrens et al., 2000)	0 (Ciarimboli et al., 2004)	**↓** (Schlatter et al., 2014)	**↓** (Guckel et al., 2012; Holle et al., 2011; Schlatter et al., 2014); V_*max*_-effect (Guckel et al., 2012)
p56^*lck*^	**↑** (Schlatter et al., 2014)	**↑** (Mehrens et al., 2000)	**↑** (Ciarimboli et al., 2004)	**↑** (Schlatter et al., 2014)	**↑** (Guckel et al., 2012; Holle et al., 2011; Schlatter et al., 2014); V_*max*_-effect (Guckel et al., 2012)
Tyrosine kinase		0 (Mehrens et al., 2000)			
cGMP		**↓** (Schlatter et al., 2002)	0 (Ciarimboli et al., 2004)		
CaM	**↑** (Schlatter et al., 2014)		**↑** (Ciarimboli et al., 2004); K_*m*_-effect	**↑** (Schlatter et al., 2014)	**↑** (Guckel et al., 2012; Holle et al., 2011; Schlatter et al., 2014); K_*m*_-effect (Guckel et al., 2012)
CamKII			**↑** (Ciarimboli et al., 2004)		
MLCK			0 (Ciarimboli et al., 2004)		
PI3K	**↑** (Schlatter et al., 2014)		0 (Ciarimboli et al., 2004)	**↑** (Schlatter et al., 2014)	**↑** (Guckel et al., 2012; Holle et al., 2011; Schlatter et al., 2014); V_*max*_-effect (Guckel et al., 2012)
Ang II				**↑** (Schlatter et al., 2014)	**↑** (Guckel et al., 2012; Holle et al., 2011; Schlatter et al., 2014); K_*m*_-effect (Guckel et al., 2012)

* Effect magnitudev depends on forskolin concentration used in (Gerlyand and Sitar, 2009): with 1 µM forskolin, no significant regulation of rOCT1 activity was observed, and with 10 µM forskolin, a significant inhibition of rOCT1 was measured.

PKA, protein kinase A; PKC, protein kinase C; p56^lck^, p56^lck^ tyrosine kinase; cGMP, cyclic GMP; CaM, Ca^2+^−calmodulin complex; CaMKII, multifunctional Ca^2+^/CaM-dependent protein kinase II; MLCK, myosin light chain kinase; PI3K, phosphoinositide 3‐kinase; Ang II, angiotensin II.

### Rapid Regulation of rOCT1

Using the same experimental approach, transport of ASP^+^ mediated by rOCT1 was demonstrated to be stimulated by PKA, PKC, CaM, and p56^lck^ tyrosine kinase. Other tyrosine kinases did not influence rOCT1-mediated transport, while cGMP inhibited it (Table 1) (Mehrens et al., 2000; Schlatter et al., 2002; Ciarimboli et al., 2005). Importantly, it was demonstrated that PKC activation directly phosphorylates rOCT1 and changes transporter affinities (Ciarimboli et al., 2005). The potential PKC-phosphorylation sites at positions S286, S292, T296, S328, and T550 seem to be important for this effect. Other studies showed a strong downregulation of rOCT1-mediated transport under PKC inhibition with staurosporine (Gerlyand and Sitar, 2009), confirming a possible PKC stimulation of rOCT1-mediated transport.

Interestingly, using tetraethylammonium (TEA^+^) as a transport tracer, PKA stimulation did not change rOCT1 activity (Gerlyand and Sitar, 2009). These results may be explained admitting that OCTs have a large binding pocket, with partially overlapping interaction domains for different substrates (Ciarimboli et al., 2005; Popp et al., 2005). Therefore, PKA may induce conformational changes in the binding domain of ASP^+^ and not in that of TEA^+^, resulting in stimulation of ASP^+^ uptake but not of TEA^+^ transport.

### Rapid Regulation of hOCT1

ASP^+^ microfluorimetry has been used also to characterize the rapid regulation of hOCT1 overexpressed in HEK293 cells or Chinese hamster ovary (CHO) cells (Ciarimboli et al., 2004). Interestingly, regulation patterns observed in the two cell systems were not different. The hOCT1 activity was downregulated by PKA stimulation. This regulation was different from what was observed for mOCT1 and rOCT1, where PKA activation stimulated the transporters (s above). Activity of PKC, myosin light chain kinase (MLCK), or PI3K did not regulate hOCT1. The p56^lck^ tyrosine kinase, CaM, and the multifunctional Ca^2+^/CaM-dependent protein kinase II (CaMKII) stimulated hOCT1 activity, similarly to what was observed for m and rOCT1. Regulation of hOCT1 by PKA and CaM was associated with changes of the transporter apparent affinity for its substrates (Ciarimboli et al., 2004).

The presence of specific phosphorylation sites in the OCT1 orthologs can probably contribute to explain the specific regulation observed in the works cited above. For example, focusing on PKA effect on mOCT1, rOCT1, and hOCT1, using the group-based prediction system GPS 5.0 (Xue et al., 2008), the same 3 potential phosphorylation sites (S334, T348, and S537) are found in the primary structure of mOCT1 (NCBI sequence NP_033228.2) and rOCT1 (UniProtKB: Q63089.1). In hOCT1 primary structure (UniProtKB: O15245.2), only one potential PKA phosphorylation site (T347) is detected. Therefore, phosphorylation of S334 and/or S537 may be responsible for PKA upregulation of mOCT1 and rOCT1 activities measured by ASP^+^ microfluorimetry.

### Rapid Regulation of ASP^+^ Transport in Freshly Isolated Mouse Proximal Tubules

Comparing acute regulation of ASP^+^ uptake in freshly isolated proximal tubules (PT) from wild-type (WT) mice and mice with genetic deletion of OCT2 (OCT2^−/−^, in these mice, the predominant OCT in proximal tubules is OCT1 (Holle et al., 2011)), an identical regulation pattern was observed. Moreover, the regulation of ASP^+^ uptake in PT isolated from WT- and OCT2^−/−^- mice was the same as that observed in HEK cells overexpressing mOCT1. These results suggest that OCT1 is the main functional OCT paralog in this part of the mouse nephron (Schlatter et al., 2014). OCT regulation by p56^lck^, PI3K, PKA, and PKC in mouse kidneys is linked to V_max_ changes of ASP^+^ transport (Guckel et al., 2012). Interestingly, the same qualitative regulation pattern of ASP^+^ transport was observed in PT from male and female OCT2^−/−^ mice. However, p56^lck^ and PKC had an approximately 20 % stronger effects in female than in male animals (Schlatter et al., 2014). A dependence of OCT-mediated transport regulation on sex has been observed also in rats (Wilde et al., 2009), probably due to a stronger endogenous expression of regulatory enzymes, such as CaM (Wilde et al., 2009).

Activation *via* p56^lck^ seems to be a rapid regulation pathway conserved along all OCT1 orthologs (s above) and the other OCT paralogs (Wilde et al., 2009; Massmann et al., 2014; Schlatter et al., 2014; Frenzel et al., 2019). Interestingly, according to the group-based prediction system GPS 5.0 (Xue et al., 2008), all OCT1 and OCT2 orthologs have a conserved potential lck phosphorylation site in the intracellular domain on tyrosine 543 (hOCT1), 545 (mOCT1 and rOCT1), or 544 (mOCT2, rOCT2, and hOCT2), close to the carboxy-terminus. The OCT3 orthologs have such a conserved potential lck phosphorylation site on tyrosine 456 (mOCT3 and rOCT3) or 461 (hOCT3) in the small intracellular loop between the transmembrane domains 10 and 11. Therefore, it can be supposed that a direct phosphorylation by p56^lck^ regulates OCT activity. Mutagenesis of these tyrosines to, for example, alanine may help to reveal whether OCT regulation by p56^lck^ is associated with phosphorylation of these sites. Such an experimental approach has been used to show that point mutations in the OCTs can have a specific influence on the binding characteristics of different substrates (Ciarimboli et al., 2005). These findings support the concept that OCTs have a large binding site with different interaction regions for diverse substrates (Koepsell, 2019; Sandoval et al., 2019). Therefore, transporter regulation studied with different OCT substrates may show different results, as outlined above for PKA regulation of rOCT1 activity.

In conclusion, the cellular processing of OCT1 may be regulated by a direct interaction with other proteins. Moreover, rapid regulation of OCT1 activity is probably necessary to adjust transporter activity to physiological requirements. This regulation is ortholog and paralog specific, and changes transport characteristics such as the affinity to known substrates and the maximum reaction velocity. Both in the liver and at least in the rodent kidneys, OCT1 mediates the first step of organic cation secretion, that is, the uptake of substrates into hepatocytes and into cells of the renal proximal tubules, respectively. For this reason, it should be investigated whether a regulation pathway able to stimulate the second step of secretion process, that is, the excretion of organic cations into the bile and urine, by, for example, the multidrug and toxin extrusion proteins (MATEs) (Kantauskaite et al., 2020), can work together with OCT regulation to globally modulated hepatic and renal substrate secretion.

## Long-Term Regulation of OCT1

### Factors Determining OCT1 Expression and Function in Humans and Mice

Tissue-specific processes activated by different pathophysiological conditions influence OCT1 expression and activity. As outlined above, OCT1 is mainly expressed in hepatocytes. However, there are few studies, which try to explain why OCT1 is highly expressed in the liver. The hepatic expression of many proteins is under the control of two transcription factors: the hepatocyte nuclear factor 4α (HNF4α) and the CCAAT/enhancer-binding protein (C/EBP) (Nishiyori et al., 1994). For this reason, the expression of mRNA coding for HNF4α and for C/EBP has been correlated with that of hOCT1 in the human liver. A significant correlation between hepatic mRNA expression of hOCT1 and that of HNF4α and C/EBP was found (Rulcova et al., 2013). Moreover, stimulation of HNF4α by dexamethasone in human primary hepatocytes increased hOCT1 expression. Two functionally important response elements for HNF4α have been found in the 5'-flanking region of the solute carrier 22A1 (*SLC22A1*) gene, the gene coding for hOCT1 (Saborowski et al., 2006; Hyrsova et al., 2016). These elements seem not to be conserved in rodents (Saborowski et al., 2006).

Another important regulator of drug transport and metabolism in humans is the hepatocyte nuclear factor 1 (HNF1), a transcription factor, which is highly expressed in the liver (Courtois et al., 1988). HNF1 has been identified as a potent regulator of hOCT1 expression. HNF1 increases *SLC22A1* promoter activity by binding to an evolutionary conserved region in intron 1 of the *SLC22A1* gene (O'Brien et al., 2013).

The presence of single nucleotide polymorphisms (SNPs) in *SLC22A1* is well known to modulate its function (Kerb et al., 2002; Shu et al., 2003; Shu et al., 2007; Tzvetkov et al., 2009; Tzvetkov et al., 2011; Tzvetkov et al., 2012; Tzvetkov et al., 2013). The SNPs of *SLC22A1* have been shown to influence hOCT1 transport characteristics (affinity K_m_ and maximal velocity V_max_) and the pharmacokinetics of drugs, which are substrates of hOCT1. This aspect of hOCT1 regulation has been already summarized in other excellent reviews (Yee et al., 2018; Zazuli et al., 2020) and will be further discussed in detail in other contributions to this special issue.

In mice, a transcriptional regulation of *Slc22a1* (murine genes are conventionally written in lowercase) by peroxisome proliferators activated receptors (PPAR) has been proposed. PPAR are transcription factors which play an important role in metabolic regulation and in determining liver function (Kersten, 2014). For example, in the liver, the nuclear receptor PPARα is activated in the fasted state, and its activation induces fatty acid oxidation and gluconeogenesis (Preidis et al., 2017). PPARγ stimulates several proteins associated with lipid uptake, triacylglycerol storage, and formation of lipid droplets (Wang et al., 2020). The physiological ligands of PPAR are fatty acids, which are mobilized during fasting or food restriction. Therefore, PPAR-α and PPAR-γ agonists are agents, which can modulate many hepatic metabolic and transport processes. In mice, feeding PPAR-α and -γ agonists increased transcriptional *Slc22a1* gene expression. In an *in vitro* model, the increased *Slc22a1* expression induced by PPAR-α and PPAR-γ agonists resulted in a stimulation of cellular organic cation uptake (Nie et al., 2005). Since OCT1 is a high-affinity choline transporter (Sinclair et al., 2000), and choline is essential for phosphatidylcholine synthesis, the stimulation of OCT1 by fatty acids may be useful to increase choline uptake when its portal blood concentrations are low (Nie et al., 2005).

There are several works demonstrating that sex can influence OCT1 expression at mRNA and protein levels. In mice and rats, renal OCT1 protein expression was higher in male than in female animals (Sabolic et al., 2011). However, renal OCT1 mRNA expression was higher in female than in male rats (Sabolic et al., 2011). Therefore, regulation of OCT1 mRNA and protein expression can be divergent, at least in rodents. It is not known whether OCT1 expression in humans is dependent on sex.

### Long-Term Effect of Kinase Activation on OCT1 Expression and Function

Regarding long-term effects of kinase activity on transporter function, 24 h incubation with 10 µM epinephrine has been demonstrated to down-regulate hOCT1 mRNA-expression *via* cAMP formation in primary human hepatocytes (Mayati et al., 2017a). These results confirm what was observed for rapid regulation of hOCT1 under stimulation of PKA activity (Ciarimboli et al., 2004), showing that this regulation axis has similar short- and long-term effects on hOCT1 function.

Long-time (24–48 h) exposure to PKC activators such as phorbol ester 12-myristate 13-acetate (PMA, 100 nM) reduced hOCT1 mRNA-expression and activity in human hepatoma HepaRG cells and primary human hepatocytes (Mayati et al., 2015). However, shorter incubation times with PMA did not change hOCT1 transport activity in HepaRG cells (Mayati et al., 2017b), confirming what was found for rapid hOCT1 regulation by PKC (s above). Therefore, it can be concluded that acute and chronic kinase activation may have also a different impact on hOCT1 activity.

How can the data be interpreted on OCT1 regulation in a physiological context? Focusing on hOCT1 and the liver, one can try to build a model integrating hOCT1 short- and long-term regulation and activation of a specific signaling pathway. For example, it is well known that activation of the cAMP/PKA pathway is important for regulation of hepatic energy metabolism. Glucagon and catecholamines stimulate in the liver the formation of cAMP and PKA, which leads to an increased glucose production, increased gluconeogenesis, and a decreased glycolysis. Activation of this pathway also influences lipid metabolism by decreasing biosynthesis of fatty acids and lipogenesis and increasing fatty acid oxidation. Moreover, activation of this pathway represses the expression of the PPAR-γ gene, which is a key regulator of lipogenic genes. Therefore, activation of the cAMP/PKA pathway leads to increased hepatic glucose production and decreased lipid accumulation (Wahlang et al., 2018). Therefore, since hOCT1 activity is rapidly inhibited by PKA activity and PPAR-γ agonists increased transcriptional *Slc22a1* gene expression, increased hepatic glucose production may be associated with immediate reduction of hOCT1 activity and decrease of hOCT1 gene expression.

## Long-Term Regulation Under Pathological Conditions and by Environmental Toxins

### Studies in Animal Models

Decreased protein expression of OCT1 in kidneys from diabetic rats was detected (Thomas et al., 2003; Grover et al., 2004), which was restored by inhibition of angiotensin-converting enzyme (Thomas et al., 2003) or insulin treatment (Grover et al., 2004). Since these changes are evident at the protein but not at the mRNA level (Grover et al., 2004), it was speculated that they are due to posttranscriptional alterations (Grover et al., 2004).

Ischemia–reperfusion injury (IRI) down-regulated mRNA and protein expression of OCT1 in rat kidneys. In this model, IRI increased NO generation by stimulation of inducible nitric oxide synthases (iNOS). Inhibition of iNOS at the end of ischemia restored OCT1 expression at normal levels, suggesting that NO is a negative regulator of OCT1 (Schneider et al., 2011).

Syngeneic and allogeneic rat kidney transplantation downregulated the mRNA and protein expression of OCT1 in the transplanted kidneys. Immunosuppression with cyclosporine A partially restored OCT1 mRNA expression in the allogeneic model (Ciarimboli et al., 2013).

Hepatic cholestasis, studied in a bile duct ligation (BDL) model in the rat, down-regulates the expression and function of rOCT1 in the liver, probably as a protection mechanism, to decrease hepatic accumulation of potentially toxic substances (Denk et al., 2004).

### Studies in Human Tissues and Human Cells in Culture

Epigenetic modifications (e.g., DNA methylation and histone modification) are heritable variations that regulate chromatin structure and DNA accessibility and can change gene expression without changing its DNA sequence. In human hepatocellular carcinoma (HCC), the mRNA and protein expression of hOCT1 were found to be significantly reduced compared with normal adjacent liver tissue (Schaeffeler et al., 2011). Methylation of *SLC22A1* seems to be associated with a lower expression of hOCT1 in HCC (Schaeffeler et al., 2011). Interestingly, the downregulation of hOCT1 in HCC has been confirmed in an independent study and was found to be associated with tumor progression and a worse patient survival (Heise et al., 2012). The same relationship between hOCT1 expression and consequences for tumor progression and patient survival has been observed in cholangiocellular carcinoma (CCA), a hepatic malignancy derived from cholangiocytes (Lautem et al., 2013). It has been observed that in diabetic patients treated only with metformin, methylation of liver *SLC22A1* decreases (Garcia-Calzon et al., 2017), suggesting that diabetes may decrease OCT1 expression by increasing *SLC22A1* methylation. However, to my knowledge, there is still no quantitative measurement of OCT1 expression in diabetic patients.

Liver function deterioration (e.g., induced by hepatitis C, primary biliary cholangitis, primary sclerosing cholangitis, alcoholic liver disease, and autoimmune hepatitis) decreased the amount of hOCT1 mRNA and protein in the liver (Drozdzik et al., 2020).

Cigarette smoking is well known to have an important pharmacological impact because it can change drug pharmacokinetics and drug–drug interactions. Cigarette smoke condensate decreases mRNA expression and activity of hOCT1 in human hepatoma HepaRG cells, probably *via* activation of the aryl hydrocarbon receptor (AhR) signaling pathway (Sayyed et al., 2016). Activation of the AhR signaling pathway may be also the mechanism by which exposure to the diesel exhaust particles (25 μg/ml, 48 h) decreases hOCT1 mRNA expression in HepaRG cells (Le et al., 2015).

Taken together, these results suggest that pathological insults and environmental toxins down-regulate OCT1 expression.

## Conclusion

In conclusion, OCT1 is subjected to a multifaceted regulation, which can change its function. Therefore, modulation of its expression and activity may have important physiological and pharmacological consequences due to the role of OCT1 for handling of endogenous and exogenous substrates such as neurotransmitters and drugs. It would be important to investigate whether and at which position OCT1 is phosphorylated by rapid regulation processes and to define the exact role of interacting proteins for transporter cellular processing. In this way, new functional meaning of SNPs in hOCT1 and/or interacting proteins can be detected. Regulation of OCT1 may change excretion of its substrates and modify toxicity of drugs and environmental toxins. Further research is necessary to clarify these important issues of OCT-mediated transport.
